# Thyroid Storm Masquerading as Multiple Organ Dysfunction Syndrome: Catch Me if You Can

**DOI:** 10.7759/cureus.39584

**Published:** 2023-05-27

**Authors:** Kashish Khurana, Sunil Kumar, Sourya Acharya, Saket Toshniwal, Nikhil Pantbalekundri

**Affiliations:** 1 Department of Medicine, Jawaharlal Nehru Medical College, Datta Meghe Institute of Higher Education and Research (Deemed to be University), Wardha, IND; 2 Department of Internal Medicine, Jawaharlal Nehru Medical College, Datta Meghe Institute of Higher Education and Research (Deemed to be University), Wardha, IND

**Keywords:** multiple organ failure, burch-wartofsky score, carbimazole, septic shock, thyroid storm

## Abstract

A 65-year-old man presented to the emergency medicine department with altered sensorium, a high-grade fever, and shock. On routine workup, he was diagnosed with acute respiratory distress syndrome with sepsis. Later, it was found that the patient had undetectable serum thyroid stimulating hormone and high triiodothyronine (T3) levels, which were diagnosed as a thyroid storm. This highlights the fact that a thyroid storm can present in any way and should be considered when determining the cause of septic shock that is not responding to standard treatment. A rare endocrine emergency, thyroid storm is a life-threatening endocrinological emergency with a considerable death rate of between 10% and 30% and multi-organ failure. It happens in thyrotoxic patients and manifests as the decompensation of several organs brought on by extreme stress. In addition to shock, the patient also had altered sensory perception, a cough, a fever, palpitations, and a sore throat. The patient was initially diagnosed with septic shock and was later treated with oral carbimazole, higher antibiotics, inotropes, and propranolol.

## Introduction

The incidence of thyroid storm, a rare metabolic disorder, is 0.2 per 100,000 annually. Thyroid hormones have an impact on every system of the body. Muscle and central nervous system (CNS) excitability are all increased by thyroid hormones, along with metabolic rate, heart rate, and ventricular contractility [1]. Thyroxine (T4) and triiodothyronine (T3), which are secreted at a ratio of 20:1, respectively, are the two main forms of thyroid hormones. The active form of T3, which has three to four times the potency of T4, is created peripherally from T4 [2].

While thyrotoxicosis refers to excessive thyroid hormone circulating for any reason, hyperthyroidism is a condition in which there is an excess of thyroid hormone that is only caused by intrinsic thyroid gland hyperfunction (including thyroid hormone overdose) [3].

A thyroid storm is the most severe form of thyrotoxicosis, and it appears as an acute, severe, life-threatening hypermetabolic state that is either brought on by excessive thyroid hormone release, which leads to adrenergic hyperactivity, or by altered peripheral thyroid hormone response in the presence of one or more precipitants [4].

Infections are the most frequent trigger for a thyroid storm. Other factors include vascular accidents, surgery, stress, parturition, eclampsia, trauma, diabetic ketoacidosis, hypoglycemia, hyperosmolar non-ketotic coma, pulmonary thromboembolism, thyroxine overdose, sudden stoppage of anti-thyroid medication, iodinated contrast medium intake, and myocardial infarction [5]. A thyroid storm may ironically develop in certain patients receiving radioactive iodine therapy for hyperthyroidism due to the discontinuation of anti-thyroid medications leading to the release of T3 and T4 from damaged thyroid follicles or the action of radioactive iodine itself. Twenty percent to twenty-five percent of the time, no acute precipitant is found [2].

## Case presentation

A 65-year-old male came to the emergency medicine department with an altered sensorium. He had also been having a fever (on presentation, the temperature was 101.4°F), cough, dyspnea, and palpitations for five days. The patient was previously healthy without any known medical conditions. The patient had no known case of thyroid disease. He was a non-alcoholic and a non-smoker. On admission, the patient was unconscious and had an increased respiratory rate. On examination, his general condition was poor. He was febrile with a temperature of 39.6°C, and his blood pressure was 80/60 millimeters of mercury. Oxygen saturation in room air was 89%, with a heart rate of 120 beats per minute. His arterial blood gas analysis revealed a pH <7.2 and a partial pressure of carbon dioxide of 62 mm of mercury.

On systemic examination, the patient had tachycardia, bilateral coarse crepitations in the lower chest, and tender hepatomegaly. On central nervous system examination, the patient was unconscious, responding to deep pain stimuli, with a Glasgow Coma Scale of 4/15, a bilateral pupil mid-dilated and reactive to light, a corneal reflex present, and a bilateral plantar reflex extensor. In view of the altered sensorium, the patient was intubated and placed on a mechanical ventilator in volume control mode.

The patient was further evaluated during his intensive care unit stay, and his laboratory investigations were suggestive of raised thyroid hormone levels, with thyroid stimulating hormone (TSH) levels being non-detectable. His platelet count was 48000/cubic millimeter, his prothrombin time was 10 seconds, and his fibrinogen levels were decreased, as shown in Table 1.

**Table 1:** The patient's laboratory investigations

Parameters	Value	Normal value
Hemoglobin	12.2	12-15 g/dL
Total leukocyte count	19500	4000-11000 cu. Mm
Platelet count	0.48	1.5- 4.1 lakhs/cubic mm
Prothrombin time	10	<3 seconds
Alkaline phosphatase	91	38- 126 U/L
Alanine aminotransferase	443	<35 U/L
Aspartate aminotransferase	856	14-36 U/L
Urea	103	15-36 mg/dL
Creatinine	1.7	0.52-1.04 mg/dL
Potassium	5.4	3.5- 5.1 meq/l
Free T3	7.95	02.77- 5.27 pg/ml
Free T4	5.72	0.78-2.19 ng/dl
Thyroid stimulating hormone	Not detectable	0.465- 4.68 micro IU/ ml
C- reactive protein	96	<1 mg/dl
International normalization ratio	2.1	<1.2
Serum ammonia	35	9-40 micro moles/L
Serum procalcitonin	20	<0.5 micrograms/ litres
Fibrinogen levels	58	>100 mg/dL

**Figure 1:**
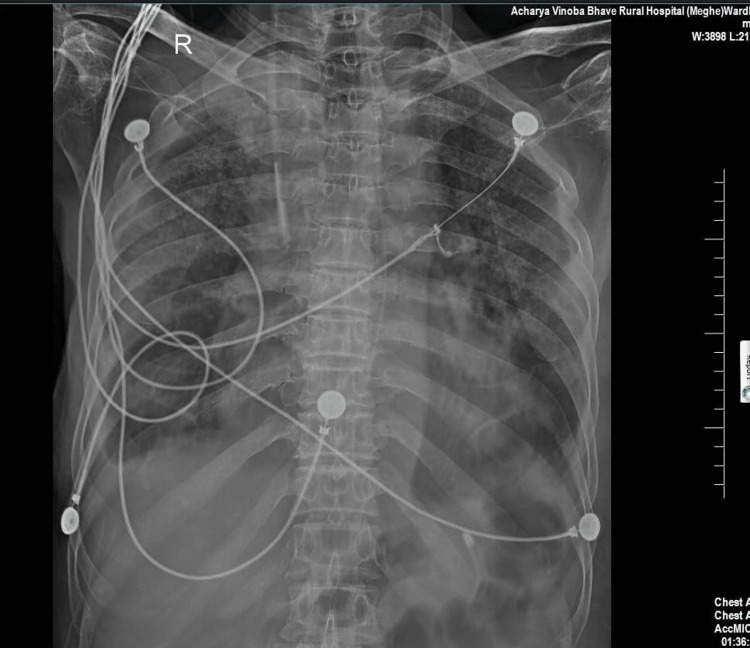
The patient's chest x-ray shows bilateral pulmonary infiltrates

His chest x-ray revealed bilateral heterogenous infiltrations suggestive of acute respiratory distress syndrome, as shown in Figure 1. 

**Figure 2:**
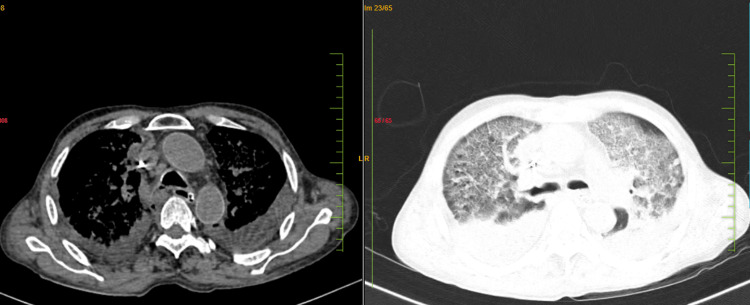
High-resolution computed tomography of the chest shows a non-specific interstitial pattern with patchy areas of consolidation noted in the right upper and left lower lobes with internal cavitations, oblique necrotic areas, and bilateral pleural effusions suggestive of acute respiratory distress syndrome

His ultrasonography of the abdomen was normal except for a grade 1 fatty liver. High-resolution CT chest revealed a non-specific interstitial pattern with patchy areas of consolidation noted in the right upper and left lower lobes with internal cavitations, oblique necrotic areas, and bilateral pleural effusions suggestive of acute respiratory distress syndrome, as shown in Figure 2.

Thyroid function tests showed thyrotoxicosis: the free T3 concentration was 7.95 (2.77 to 5.27 pg/ml), the free T4 concentration was 5.72 (0.78-2.19 ng/dl), and thyroid stimulating hormone was not detectable (ref. range 0.465-4.68 micro IU mL). His 2D echocardiogram was suggestive of a low left ventricular ejection fraction of 35%. According to the Burch-Wartofsky scoring or Akamizu's diagnostic criteria, the patient’s score was 70, which was highly suggestive of a thyroid storm. The patient was started on ionotropic support with injectable dobutamine (10 micrograms/kg/minute), injectable vasopressin (2.4 ml/hour), and injectable norepinephrine (1 microgram/kg/minute). The patient was given 100 mg of hydrocortisone intravenously thrice a day and broad-spectrum antibiotics like injectable piperacillin-tazobactam (4.45 grams intravenously thrice a day) and injectable meropenem (1 gram intravenously twice a day). The heart rate was controlled with a tablet of propranolol (40 mg) taken thrice a day orally. Carbimazole (20mg) was started three times a day. The patient was weaned from mechanical ventilation after eight days and recovered without any neurological deficit. The sensorium improved on the seventh day. The patient was shifted from the medicine ICU after nine days to the medicine ward and eventually discharged on a tablet of carbimazole (20 mg thrice a day). On follow-up after one month, the patient was doing well.

## Discussion

The cytoplasm of cells absorbs circulating T4 and T3 in situations with increased circulating thyroid hormone. The 5′-deiodinase enzyme deiodinates the outer ring of T4 to produce T3, which is its active form. T3 then activates genes in the cytoplasm by entering the nucleus, where it binds to thyroid hormone receptors or thyroid hormone-responsive components [2].

During thyrotoxicosis, thyroid hormone levels are raised, which, through a negative feedback mechanism, inhibits thyroid stimulating hormone (TSH). By releasing thyroid hormones from their binding sites, increasing the sensitivity of the tissues' receptors through an increase in the density of adrenergic receptors on target cells, or altering signaling pathways post-receptor, precipitants like infection, stress, myocardial infarction, or trauma will increase the effects of thyroid hormones when a thyroid storm occurs [3]

Thyroid storm that, despite early detection and intensive resuscitation, had a fatal outcome that included multiple organ failure syndrome, diffuse intravascular coagulation (DIC), and death [4]. In our case, early diagnosis and intervention led to total improvement.

A thyroid storm with DIC has been recorded in a very small number of instances. Burch et al. established the internationally accepted diagnostic criteria for thyroid storm; a score of > 45 indicates thyroid storm [5]. Our patient's score was 70. Our patient met these requirements, having increased FT3, FT4, and two of the five symptoms. In our patient, the cause of the thyroid storm was multifactorial, including acute-onset congestive failure, medical non-compliance, and/or clinical palpation of his thyroid gland [6]. Thyroid storms can also present as hyperemesis gravidarum and neuropsychiatric manifestations [7,8].

In rural areas where intravenous antithyroid drugs are not readily available, early diagnosis and treatment with oral medication can reduce morbidity and mortality. Without therapy, thyroid storm mortality can range from 80% to 100%; however, with medication, mortality can range from 10% to 50% [7]. Therefore, early diagnosis of a thyroid storm is essential to reducing the morbidity and mortality of this disorder. The diagnostic approach for thyroid storm may use Akamizu's diagnostic criteria or Burch-Wartofsky score [5]. It is a medical emergency that frequently occurs in conjunction with a triggering sickness and mimics sepsis and septic shock due to multiple organ failure [5]. To reduce mortality and morbidity, septic shock, a multisystem disease that can coexist with thyroid storm and present similarly, needs to be diagnosed as soon as possible.

## Conclusions

This case highlights that, though multiple organ dysfunction syndrome (MODS) is a well-known and well-described clinical complication in the ICU, hyperthyroidism in the form of a thyroid storm as its cause is frequently missed, or there is a delay in diagnosis due to conflicting signs and symptoms. A thyroid storm is precipitated by an acute critical condition, which is usually multifactorial. A thyroid storm is a rare medical emergency with a sudden onset, rapid progression, and high mortality. Early diagnosis based on the criteria and treatment with antithyroid drugs like carbimazole or propylthiouracil in the initial stages is crucial for survival.
